# Lingering Sound: Event-Related Phase-Amplitude Coupling and Phase-Locking in Fronto-Temporo-Parietal Functional Networks During Memory Retrieval of Music Melodies

**DOI:** 10.3389/fnhum.2019.00150

**Published:** 2019-05-22

**Authors:** Yi-Li Tseng, Hong-Hsiang Liu, Michelle Liou, Arthur C. Tsai, Vincent S. C. Chien, Shuoh-Tyng Shyu, Zhi-Shun Yang

**Affiliations:** ^1^Department of Electrical Engineering, Fu Jen Catholic University, New Taipei City, Taiwan; ^2^Institute of Statistical Science, Academia Sinica, Taipei, Taiwan; ^3^Department of Psychology, National Taiwan University, Taipei, Taiwan; ^4^Max Planck Institute for Human Cognitive and Brain Sciences, Leipzig, Germany

**Keywords:** EEG, musical delayed match-to-sample, phase-locking value, event-related phase-amplitude coupling, memory retrieval

## Abstract

Brain oscillations and connectivity have emerged as promising measures of evaluating memory processes, including encoding, maintenance, and retrieval, as well as the related executive function. Although many studies have addressed the neural mechanisms underlying working memory, most of these studies have focused on the visual modality. Neurodynamics and functional connectivity related to auditory working memory are yet to be established. In this study, we explored the dynamic of high density (128-channel) electroencephalography (EEG) in a musical delayed match-to-sample task (DMST), in which 36 participants were recruited and were instructed to recognize and distinguish the target melodies from similar distractors. Event-related spectral perturbations (ERSPs), event-related phase-amplitude couplings (ERPACs), and phase-locking values (PLVs) were used to determine the corresponding brain oscillations and connectivity. First, we observed that low-frequency oscillations in the frontal, temporal, and parietal regions were increased during the processing of both target and distracting melodies. Second, the cross-frequency coupling between low-frequency phases and high-frequency amplitudes was elevated in the frontal and parietal regions when the participants were distinguishing between the target from distractor, suggesting that the phase-amplitude coupling could be an indicator of neural mechanisms underlying memory retrieval. Finally, phase-locking, an index evaluating brain functional connectivity, revealed that there was fronto-temporal phase-locking in the theta band and fronto-parietal phase-locking in the alpha band during the recognition of the two stimuli. These findings suggest the existence of functional connectivity and the phase-amplitude coupling in the neocortex during musical memory retrieval, and provide a highly resolved timeline to evaluate brain dynamics. Furthermore, the inter-regional phase-locking and phase-amplitude coupling among the frontal, temporal and parietal regions occurred at the very beginning of musical memory retrieval, which might reflect the precise timing when cognitive resources were involved in the retrieval of targets and the rejection of similar distractors. To the best of our knowledge, this is the first EEG study employing a naturalistic task to study auditory memory processes and functional connectivity during memory retrieval, results of which can shed light on the use of natural stimuli in studies that are closer to the real-life applications of cognitive evaluations, mental treatments, and brain-computer interface.

## Introduction

With an increasing interest in neural mechanisms of music perception and naturalistic experimental stimulation over the past decade, the neural mechanisms underlying auditory memory have received substantial attention in the field of neuroimaging (Weinberger, 2014). Unlike visual working memory, neurodynamics and brain functional connections of auditory working memory have not been well studied. Studies recording brain activation using modalities such as electroencephalography (EEG), magnetoencephalography (MEG), and functional magnetic resonance imaging (fMRI) have explored the cognitive processes of auditory memory with distinct temporal and spatial scopes according to the resolution of these neuroimaging modalities (Zatorre et al., 1994; Schulze et al., 2011; Albouy et al., 2013, 2015, 2017; Huang et al., 2013; Plakke and Romanski, 2014; Kumar et al., 2016; Wilsch and Obleser, 2016). In the present study, high temporal-resolution EEG signals were recorded and two indices, phase-amplitude coupling (PAC) and phase-locking, were utilized to evaluate the neural dynamic and functional connectivity between brain regions during an auditory working memory task. The PAC is an index that reflects the modulation of low-frequency phases on high-frequency amplitudes of EEG signals during memory or other cognitive processes, and it is frequently observed in hippocampal regions (Axmacher et al., 2010). The phase-locking is an index of functional connectivity and provides a quantitative measure of the phase relationships between EEG signals obtained from two distinct electrodes or reconstructed brain sources. Although phase-locking has been considered important in studying memory processes (Fell and Axmacher, 2011), most of the previous studies have focused on the brain connections in visual modality (Klimesch, 1999; Knyazev, 2007; Düzel et al., 2010). Through clarifying the intra- and inter- regional brain activities, the aim of this study is to address the following issues: (1) the existence of PAC in the neocortex as an indicator of neural coding scheme formed by EEG oscillations during auditory working memory processes; (2) the brain regions involved in auditory working memory and their functional connections; and (3) the temporal dynamics of functional connections during auditory memory retrieval. By verifying the above hypotheses, EEG dynamics could be viewed as promising markers of auditory working memory and the level of PAC and phase-locking would fluctuate in accordance with different processing stages, such as memory retrieval. Based on previous findings on auditory working memory, we proposed a novel approach using musical stimuli (i.e., melodies), which are closer to daily-life experiences, to study EEG dynamics during working memory processes. The design of a real-life and unpredictable paradigm could minimize the bias in research results on memory processing caused by the changes in slow brain rhythms possibly generated by a predictable paradigm (Schroeder and Lakatos, 2009).

### Neuromusic and Auditory Memory

It has been proposed that tonal or musical stimuli can induce shared or distinct activities among brain regions and functional networks as compared with verbal stimuli, language, or other types of auditory cognition (Koelsch et al., 2009; Schulze et al., 2011). This emerging research field – neuromusic – has focused on issues including the perception of acoustic features (Cong et al., 2014), the emotional impact of music (Lin et al., 2010), auditory sensorimotor integration (Mathias et al., 2016), and music training. Most of these complex processes can be related to one rather basic cognitive function: working memory. The precise role of working memory, however, is still debated, and the existing literature has emphasized mainly on visual processes, rather than auditory modalities including tones, chords, complex melodies, or even verbal and language stimuli. Furthermore, experimental designs with music stimuli close to real-life experiences are scarce. Therefore, to elucidate the neural mechanisms contributing to musical experiences, it is of great interest to incorporate innovative tasks and neuroimaging protocols.

Over the past few years, a growing body of studies has explored the neural mechanisms underlying auditory working memory (Huang et al., 2013; Plakke et al., 2013; Plakke and Romanski, 2014; Munoz-Lopez et al., 2015). Several imaging studies have addressed the functional neuroarchitecture of one unique component of the auditory memory: tonal working memory. For example, Schulze et al. (2011) presented sequences of spoken syllables and sine wave tones simultaneously to participants and asked them to rehearse syllables or tones separately under different task conditions. They found that several cortical regions, including the Broca’s area, premotor cortex, supplementary motor area (SMA), left insular cortex, and inferior parietal lobe, were involved in both tonal and verbal rehearsals. However, there was increased activation in some subcortical structures, including the right globus pallidus and right caudate nucleus, only during the rehearsal of tonal working memory (Koelsch et al., 2009). Additionally, blood oxygen level-dependent (BOLD) signals in a network across the frontal, parietal, and temporal cortices have been shown to be elevated during the course of tonal working memory tasks, such as musical congruence tasks (Groussard et al., 2010) and musical n-back tasks (Pallesen et al., 2010). Among different brain structures, the frontal cortex is considered to play a crucial role not only because it has massive connections with other components of the tonal working memory circuits, but also because it represents and coordinates key information for auditory memory recognition in primates (Plakke et al., 2013; Plakke and Romanski, 2014). Recently, Burunat et al. (2014) reported a genuine working memory task with a musical piece (i.e., *Adios Nonino*) as the stimulus. They found that there were strong connections among the dorsolateral prefrontal cortex (DLPFC), SMA, cerebellum, and hippocampus when participants identified the small segments of the musical piece embedded in an auditory stream (expected to trigger tonal working memory). Collectively, these studies addressed the key components of the circuits contributing to auditory working memory – especially for tonal stimuli. Further investigations are in need to elucidate the circuits and their underlying neurodynamics.

### Neuroimaging Findings in Auditory Working Memory

Consistent with the fMRI findings, studies using source reconstruction protocols with EEG and MEG data have also revealed that similar brain structures are engaged in auditory working memory tasks. Taking advantage of the high temporal and spatial resolution of MEG data, Nolden et al. (2013) showed that the inferior frontal gyrus, superior temporal gyrus, and parietal structures are involved in the retention of simultaneously presented tones, with their activation increased as the number of sounds retained in the auditory working memory increased. More importantly, in these structures, oscillatory activities in the alpha (8–12 Hz) and gamma (30 Hz and above) frequency bands were found to be related to auditory working memory load (Wilsch and Obleser, 2016). The power of alpha oscillations in the posterior parietal cortex, right superior parietal cortex, right SMA, and superior temporal gyrus has been reported to be positively correlated with the number of retained items (Leiberg et al., 2006; Obleser et al., 2012), and it decreases as the load dissolves (Wilsch et al., 2014; Wöstmann et al., 2015).

Further, the existing literature has suggested that theta synchronization is an indicator of visual and auditory working memory (Knyazev, 2007; Düzel et al., 2010; Albouy et al., 2017). The encoding of new information might be reflected by theta oscillations in hippocampo-cortical feedback loops (Klimesch, 1999). For the maintenance of memory, delta and theta oscillations were discovered to be increased with greater connectivity in the frontal and parietal regions (Toth et al., 2012). There have also been studies finding that the retrieval and formation of memory are associated with theta synchronization and phase-locking in the prefrontal and mediotemporal regions (Fell et al., 2008; Fell and Axmacher, 2011). Recently, theta synchronization and phase-locking between the frontal and parietal regions have been found to be correlated with auditory working memory performance (Albouy et al., 2017). Different from previous results regarding visual or auditory working memory using single tone as stimuli, the results of our study propose a relationship between natural and musical working memory processes and brain oscillations with more complex musical sequences and closer to real-life experiences.

### Functional Connectivity, Phase-Amplitude Coupling, and Working Memory

The relationship between visual working memory and brain connections has been widely studied over the past decade. Using EEG, fronto-parietal communication via theta and alpha coherence has been observed in visuospatial working memory tasks (Sauseng et al., 2004, 2010). Further, from the viewpoint of theta–gamma coding schemes (Lisman and Buzsáki, 2008), the amplitude of gamma oscillations is modulated by the phase of theta oscillations during the encoding stage of working memory processes. In a series of subsequent studies, the intra-regional PAC between low-frequency theta/alpha and high-frequency gamma oscillations has been identified in the encoding and retrieval of visual memory processes in both the hippocampus of rats (Tort et al., 2009, 2010) and the parietal cortices of humans (Tzvi et al., 2016). However, unlike visual processes, functional connectivity and neurodynamics related to auditory working memory have not been well established. Recent studies have mainly focused on the network dynamics between the frontal and temporal regions during auditory oddball tasks. Hsiao et al. (2010, 2014) proposed that the fronto-temporal network exhibited phase synchronizations in theta and alpha bands during the detection of auditory mismatches. Additionally, Choi et al. (2013) utilized two methods, phase synchrony and bidirectional Granger causality, to demonstrate a reliable brain connectivity between the frontal and temporal areas in the theta band during the processing of auditory deviations, supporting the idea that frontal and temporal interactions are important during auditory deviant processing (Lin et al., 2007; Abrams et al., 2013; Choi et al., 2013; Hsiao et al., 2014; MacLean and Ward, 2014). Furthermore, slow rhythms, such as theta, have been suggested to be essential in frontotemporal networks, consistent with the previous work claiming the stronger theta oscillations during attentional control processes and the maintenance of short-term memory information (Klimesch et al., 2008). These neurodynamics observed in different brain regions could be seen as the supporting evidence of neural communication and spike time-dependent plasticity, which are correlated with working memory, long-term memory and their interactions (Fell and Axmacher, 2011).

### Importance of Natural Stimulation

These slow rhythms have been reported to be essential in memory-related cognitive tasks, and its engagement could be varied across experimental designs. Stimuli in memory tasks can be divided into two categories according to their interval timing: rhythmic or continuous modes of operation (Schroeder and Lakatos, 2009). The design of continuous or predictable paradigms in some previous studies might have resulted in changes in low-frequency oscillations, thus biasing research results on memory processing (Schroeder and Lakatos, 2009). Hence, interval timing tasks have been argued to result in altered brain activities when studying memory mechanisms (van Rijn, 2016). The utilization of natural, unpredictable, or naturalistic stimuli has thus become a growing trend in neuroscience for measuring responses reliably since complex behaviors are believed to be expressed only within real-life natural contexts (Hasson et al., 2010). Previous studies have addressed the natural stimulation with different types of brain imaging modalities. These real-world oriented tasks, such as natural vision when watching a movie (Betti et al., 2013), story listening (Brennan et al., 2012), hybrid natural-artificial stimuli with an MTV-style clip (Carmi and Itti, 2006), natural auditory scenes (Chandrasekaran et al., 2010), and natural conditions of judgment (Dastjerdi et al., 2013), would be more complex than traditional trial-based tasks. Specific brain networks associated with these naturalistic stimuli were discussed (Golland et al., 2007), and the evaluation of response reliability was also considered due to the larger inter-group differences in these complex stimuli (Hasson et al., 2010). Under an auditory modality, since highly artificial auditory stimuli and tasks can yield results that are unrepresentative of how brains operate in the real world, recent studies have started to use naturalistic stimuli to examine the integration of complex auditory sequences (Abrams et al., 2013; Burunat et al., 2014). Abrams et al. (2013) reported naturalistic and real-world music stimuli with greater BOLD responses across listeners in certain brain regions, such as the primary auditory and auditory association cortex, right-lateralized structures in the frontal and parietal cortices, and motor planning regions, compared to pseudo-musical control conditions. Burunat et al. (2014) later introduced an fMRI experiment with naturalistic music to explore the brain activities underlying working memory in 2014, and they located certain areas related to auditory working memory.

In this study, we investigated the EEG oscillatory activity and functional connectivity of auditory working memory with naturalistic musical stimuli. We employed a musical delayed match-to-sample task. Participants were instructed to listen to musical melodies and to decide whether the current segment was identical or only similar to the previously played target melodies. In addition to brain oscillations, intra-regional PAC between low- and high-frequency bands and inter-regional phase-locking values (PLVs) were computed to further evaluate the auditory memory processes. First, we provide details about the participants, experimental procedures, and analysis methods in the next section. Then, the analysis results regarding behavioral responses, brain oscillations, PAC, and phase-locking during natural musical memory retrieval are reported. Finally, we discuss our experimental findings regarding auditory memory processes and our hypothesis about the existence of inter- and intra- regional connections during auditory memory retrieval. The presence of PAC during memory retrieval and brain connections were demonstrated to exist and were highly dynamic during the cognitive processing of auditory memory. To the best of our knowledge, this study is the first EEG study employing a naturalistic task to study auditory memory processes and functional connectivity during memory retrieval.

## Materials and Methods

### Participants

Thirty-six healthy right-handed participants were recruited for the experiment. Prior to the experiment, all of the participants were paid $17 and asked to provide informed consent in accordance with the procedures of the human subject research ethics committee/Institutional Review Board (IRB) at the Academia Sinica, Taiwan, and then to complete a rating scale to estimate their familiarity with distinct types of music. The participants were instructed to report their preferences in music types, such as popular, rock, jazz, country, or classical music. None of the participants had showed preference of the music type and reported familiar with the soundtrack used as experimental stimuli. Data of 6 participants with task accuracy less than 70% were rejected. Additionally, for functional connectivity analysis, the dipole sources in the frontal, central, temporal, and parietal regions needed to be identified for each participant. Therefore, 10 participants were excluded from further analysis due to loose dipole fitting (see section “Clustering of Independent Components” for more details). Consequently, a total of 20 participants (10 female) with a mean age of 27.4 ± 5.60 years old were included in the data sample.

### Experimental Paradigm

**FIGURE 1:**
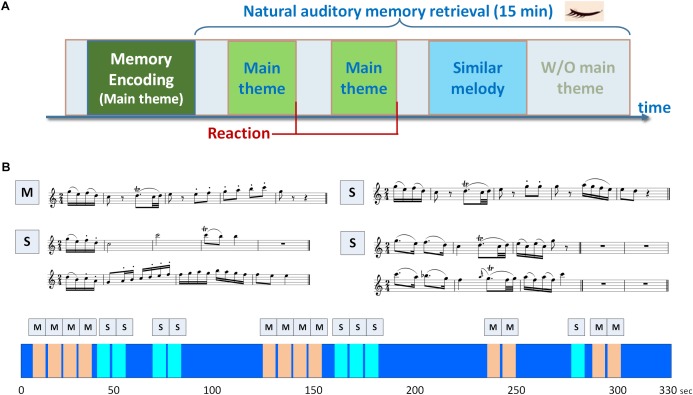
Experimental paradigm of the musical delayed match-to-sample task. **(A)** The task consisted of an encoding phase and a retrieval phase. During the encoding phase, the participants were presented with a soundtrack, in which a 3,896-ms main theme was repeated for five times with a 4,000-ms silent inter-theme interval. The participants were instructed to hold the details of the main theme in their minds. In the retrieval phase, the participants were instructed to press a key as soon as they identified the main theme in a 15-min soundtrack. **(B)** Two categories of memory items were designed in the paradigm, including the main theme (labeled as M) and three types of similar melodies (labeled as S). The similar melodies in the soundtrack were designed with the beginning 1950- or 475-ms identical to the main theme. The sequence of main theme and similar melodies in the soundtrack is shown in the lower portion of **(B)**, and the soundtrack was repeated for three times.

A musical delayed match-to-sample task (DMST) with melodies as experimental stimuli was designed for examining the brain oscillations and functional connectivity underlying auditory working memory. As shown in Figure 1A, the task consisted of an encoding phase and a retrieval phase. During the encoding phase, the participants were presented with a 40-s soundtrack, in which a 3,896-ms main theme (i.e., the *target*) was repeated five times with a 4,000-ms silent inter-theme interval. The participants were instructed to recognize the main theme and to hold its details in their minds. In the retrieval phase, a new 15-min soundtrack was introduced (Figure 1B), composed of the main theme presented before the encoding phase and three types of similar melodies (i.e., the *distractors*), and the participants were instructed to press a key as soon as they identified the main theme. The order of the main theme and similar melodies were shown in the lower portion of Figure 1B, and the sequence was the same for all the participants. The main theme and the distractors were identical in the beginning 475- or 1,950-ms time window, and all of these melodies were extracted from a rondo movement from Mozart’s Oboe Concerto in C major. The numbers of trials for the encoding phase, targets, and distractors were 5, 36, and 24, respectively. The number of trials for the encoding phase was five to ensure that the participants were able to remember the main theme before the onset of the retrieval phase. Since the experimental paradigm is a naturalistic classical soundtrack, the numbers of trials for the targets and distractors were 36 and 24 according to the original structure of the melodies. No additional modification was made to the original soundtrack. Only the target and distractor trials in the retrieval phase were included in further analysis due to the unbalanced trial numbers in this experimental design using natural stimuli. A training session with a different soundtrack was performed to ensure that the participants were familiar with the task goal, and were able to identify the main theme. In the training session, the main theme was repeated for five times in the encoding phase, and the participants were then instructed to press a key immediately after they recognized the main theme in a 3-min soundtrack. The training session was repeated and could only be finished if the participant correctly recognized the main theme. All the stimuli were programmed using Psychophysics Toolbox Version 3 (Brainard, 1997; Pelli, 1997; Kleiner et al., 2007) and MATLAB R2013b (The MathWorks, Inc., Natick, MA, United States) software to control the duration and sequence of soundtracks, and to send trigger signals during EEG recording. The sound stimuli were displayed by a speaker with volume adjusted by the participants to a comfortable level. All participants sat in a soundproof chamber, and the EEG signals were recorded throughout the experiment. To exclude visual artifacts, the participants were asked to keep their eyes closed, and all of the instructions and task stimuli were delivered using speakers.

### EEG Recording and Data Analysis

Electroencephalographys were recorded with an electrode cap (Quik-Cap128 NSL, NeuroScan, Inc., Charlotte, NC, United States) from 132 scalp locations, including 122-channel EEG, vertical and horizontal electrooculography (VEOG and HEOG), bipolar electrocardiography (ECG), and bipolar electromyography (EMG) electrodes, along with six facial-muscle channels. The electrode impedance was kept at less than 5 kΩ. Reference and ground electrodes were placed at Cz and FzA according to the 10–20 system. Signals were digitized at a sampling rate of 1 kHz and were amplified using two Synamps 64-channel amplifiers (NeuroScan, Inc., Charlotte, NC, United States) with 0.1–50 Hz analog bandpass filtering. The recorded EEG signals were re-referenced to an average reference by subtracting the mean activity across channels from each channel. Artifacts, such as eye movements, blinks, muscle tension, and line noise, were estimated with an independent component analysis (ICA) procedure, based on the reference signals in the VEOG, HEOG, ECG, and EMG channels (Jung et al., 2000). Independent components (ICs) with significantly higher correlations (*R*^2^ > 0.9) with the reference signals were then removed from the raw EEG data. For time-frequency analysis, the corrected continuous data were segmented into epochs of −800 to 5,000 ms following the stimulus onset, and the mean activities within the pre-stimulus interval served as the baseline. The ICs were first clustered to dipoles with source locations. Time-frequency analysis was then applied to evaluate if there were brain oscillations in different frequency bands. The phase-amplitude coupling within each single source dipole related to musical working memory was then observed between the low-frequency phase and high-frequency amplitudes. Finally, the results of phase-locking, which is an index of functional connectivity, revealed connections between brain regions in distinct frequency bands which can form the temporal dynamics of the fronto-temporo-parietal network.

### Clustering of Independent Components

The DIPFIT2 plug-in (Acar and Makeig, 2010) in EEGLAB toolbox (Makeig, 1993; Delorme and Makeig, 2004) was used to solve the inverse problem of EEG signals from sensors to source levels. The ICs obtained from ICA were applied to the DIPFIT2 to locate the source dipoles. Components with equivalent dipoles located outside of the modeled brain volume and residual variance larger than 20% (Iversen and Makeig, 2014) were excluded. A standardized boundary element head model (BEM) (Oostenveld and Oostendorp, 2002) was used for head modeling in advance. After the source modeling by dipole fitting, group analysis was performed by registering the individual data to a brain template provided by the Brain Imaging Center, Montreal Neurological Institute (Collins et al., 1994). The locations were therefore co-registered and mapped to a 3D brain image. According to their dipole locations and spectral characteristics, the EEG source components from all of the participants were then clustered into five regions: the frontal, central, bilateral temporal, and parietal areas. In this study, the dipole sources from these five regions were required to be identified from each participant to conduct further analysis of functional connectivity. However, previous EEG studies using ICA and dipole fitting (Onton et al., 2006; Lin et al., 2014; Chou et al., 2015) mentioned that some participants may lack one or more independent components after component selection during clustering analysis. Therefore, participants with loose dipole fitting of ICs from these regions were excluded from further analysis. The time-frequency analysis, phase-amplitude coupling, and phase synchronization described in the following subsections utilized the source information of these regions defined by the ICs and dipole fitting to evaluate brain oscillations and functional connectivity.

### Time-Frequency Analysis

Using the EEGLAB toolbox (Makeig, 1993; Delorme and Makeig, 2004), the epoched EEG data first underwent Morlet wavelet transformation to retrieve the event-related spectral perturbations (ERSPs) in each trial. For the clustered EEG sources located in the five regions, averaged ERSPs were then calculated according to the baseline (800 ms before the onset of stimuli). Since the reaction time to mark the presentation of the main theme varied slightly across participants, the ERSPs were therefore time-warped to the mean reaction time among all of the participants. The time-frequency signals were dilated or compressed to have identical time lengths after time-warped to enable group analysis (Gwin et al., 2011). For each time-frequency interval, the parametric paired *t*-test was conducted to examine the difference between the two experimental conditions: the target with main theme versus the distractors with similar melodies in the retrieval phase.

### Event-Related Phase-Amplitude Coupling

Several methods have been proposed to be able to detect the strength of phase-amplitude coupling, such as the modulation index, correlation coefficient, and generalized linear model (Penny et al., 2008; Tort et al., 2010). These methods have been widely used in recent years, and each of them has its pros and cons in managing different types of data (Tort et al., 2010). However, there are two common drawbacks to these traditional methods: the loss of temporal resolution and the requirement of a pre-defined time window. To address these issues, event-related phase-amplitude coupling (ERPAC) was reported (Voytek et al., 2013), which is a modification of the correlation coefficient method (Penny et al., 2008). It calculates the strength of phase-amplitude coupling over trials, instead of time points, and the variation in phase-amplitude coupling can therefore be detected over a short time period.

In this study, we utilized the ERPAC method and measured the strength of coupling at each time point from each trial of the musical DMST. According to previous EEG and MEG studies, the PAC phenomenon has consistently been observed in the frontal and parietal regions during cognitive tasks (Friese et al., 2013; Arnal et al., 2014; Roux and Uhlhaas, 2014; Rajji et al., 2016; Tzvi et al., 2016). Hence, two components from the frontal and parietal regions were utilized in each participant for further analysis of PAC levels. The instantaneous phases and envelopes of amplitudes from each component were first calculated by Hilbert transform (HT). We denote a pair of bandpass filtered EEG time series in a frequency range of interest as *X_p_*(t) and *X_q_*(t) at time *t*. The analytic form of the *p*th time series can be obtained by the HT as follows:

$$Z_{p}(t)=X_{p}(t)+\mathrm{jHT}(X_{p}(t))=A_{p}(t)e^{i\phi_{p}(t)},\text{ where}$$

$$j=\sqrt{-1},\;A_{p}(t)=\sqrt{X_{p}^{2}(t)+\mathrm{HT}(X_{p}(t))},\text{ and}$$

$$\mathrm{HT}(X_{p}(t))=\frac{1}{\pi }P.V.\int_{-\infty }^{\infty }\frac{X_{p}(\tau )}{t-\tau }d\tau ,$$

where P.V. indicates the integral taken in the sense of the Cauchy principal value. The instantaneous phase is defined as

$$\phi_{p}(t)=\mathrm{artan}\left(\frac{\mathrm{HT}\left(X_{p}(t)\right)}{X_{p}(t)}\right),$$

with ϕ_*p*_ (t) ∈ [−π, π). Pearson’s correlation between the phase of the *p*th time series, ϕ_*p*_ (t), and the envelope of amplitudes of the *q*th time series, *A_q_* (*t*), could be computed in the following:

$$r=\mathrm{Corr}\left(\phi_{p}(t),\;A_{q}(t)\right)=\frac{\mathrm{cov}\left(\phi_{p}(t),\;A_{q}(t)\right)}{\sigma_{\phi_{p}(t)}\sigma_{A_{q}(t)}},$$

where *cov*(ϕ_*p*_ (*t*), *A_q_* (*t*)) denotes the covariance between ϕ_*p*_ (*t*) and *A_q_* (*t*), and σ_ϕ_*p*_(*t*)_ and σ_*A*_*q*_(*t*)_ are the standard deviations. Note here that *p* and *q* could be EEG signals from the same or different sources. The amplitude-co-modulated or amplitude-normalized envelope-to-signal correlation was defined in Penny et al. (2008) to calculate the correlation between *cos*(ϕ_*p*_ (*t*)) and *A_q_* (*t*). It could be defined as an amplitude-normalized correlation,

$$r_{\mathrm{ca}}=\mathrm{Corr}\left(\cos\left(\phi_{p}(t)\right),\;A_{q}(t)\right).$$

Since *r_ca_* is unable to detect the coupling at 1/4 or 3/4 of *X_p_* (t) (Cohen, 2008) (e.g., cos((1/4)2π) = 0), circular-linear correlation (ρ) (Berens, 2009) was used to fix the problem. The method utilizes circular-linear correlation, which linearizes the phase variable into its sine and cosine components and calculates a correlation coefficient, ρ, such that

$$\rho =\sqrt{\frac{r_{\mathrm{ca}}^{2}+r_{\mathrm{sa}}^{2}-2r_{\mathrm{ca}}r_{\mathrm{sa}}r_{\mathrm{cs}}}{1-r_{\mathrm{cs}}^{2}}}$$

where *r_sa_* = *Corr*(sin (ϕ_*p*_ (*t*)), *A_q_* (*t*)) and *r_cs_* = *Corr* (sin (ϕ_*p*_ (*t*)), cos (ϕ_*p*_ (*t*))).

The ERPAC method is applied by taking the frequency-dependent instantaneous amplitude of each independent component as the target and the sine and cosine components of the phase as the regressors. The correlation between the amplitude of one EEG source (*A_q_*) and the phase of the other EEG source (ϕ_*p*_) is calculated at each time point. Therefore, the method is event-related, and the phase-amplitude coupling is calculated at each time point across trials. To evaluate cross-frequency ERPAC, the time course of a component was first bandpass filtered with a narrow-band convolution filter centered at a specified frequency with a bandwidth of 2 Hz. The ERPACs were calculated at every frequency bin from 2 to 50 Hz across trials and at each time point. The ERPACs from each component dipole selected from the frontal and parietal regions were then averaged over every 300 ms for each epoch during auditory memory retrieval or under the condition of distractor trials. The window size of 300 ms was defined larger than one low-frequency theta cycle and contained at least one theta wave for the analysis.

The ERPAC significance can be obtained by the comparison between the correlation coefficients (ρ) of the EEG component and the surrogate data distribution. The signal of each independent component was randomly permuted to generate surrogate data. In the initial stage, the frequency ranges of phase and those of the amplitude were selected by comparing the ERPAC values in the selected ranges with ten surrogate data. Each significant level from cross-frequency ERPACs between 2 and 50 Hz was analyzed, respectively, for each time period. Frequency bands with pronounced ERPACs are defined as the frequency bands of interest. After the frequency ranges were decided, the surrogate distribution was constructed using 500 random ERPAC values. The significance *p*-value associated with an observed ERPAC value was computed using a surrogate test by comparing the value with the surrogate distribution.

### Phase-Locking Value

The interaction between distinct brain regions can be represented by the phase or amplitude correlation of the recorded EEG signals. Among the many estimations of functional connectivity between two time series, PLV, phase lag index (PLI), and coherence are widely used and have been considered robust indices (Lachaux et al., 1999; Sauseng et al., 2005, 2008; Stam et al., 2007). Coherence is calculated from cross-spectral density, which is a spectral measure of correlation between EEG signal pairs. PLV and PLI both perform better in detecting the true changes of synchronization than coherence (Stam et al., 2007). Unlike coherence, PLV can provide quantitative phase relationships between two brain signals which has been reported an important index in memory processes (Fell and Axmacher, 2011). Phase synchronization or phase-locking is defined as the locking of the phases for each pair of channels, independent components, or dipoles. The instantaneous phase of the *k*th trial from two filtered signals, *X_p_*(t) and *X_q_*(t), would first be calculated as $\Phi_{p}^{k}\left(f_{m},t\right)$ and $\Phi_{q}^{k}\left(f_{n},t\right)$, which are defined by Hilbert transformation in the previous subsection. The frequency relationship of the two signals is *m* : *n*. Since

$$\frac{m+n}{2\cdot n}\cdot f_{n}=\frac{n+m}{2\cdot m}\cdot f_{m},$$

the phase difference of this pair of signals is

Δϕpqk(fm,fn, t)≅(n+m2⋅mϕpk(fm, t)−m+n2⋅nϕqk(fn, t)) modulus 2π.

The PLV for each single trial with an equal time length is defined by

$${\hat{\Gamma }}_{\phi }\left(f_{m},f_{n},\;t\right)=\left|\langle e^{j\cdot \Delta \phi_{k}(f_{m},f_{n},t)}\rangle \right|,$$

and the PLV for the total *K* trials from a pair of components at time *t* is defined as follows:

$$\mathrm{PLV}\left(f_{m},f_{n},\;t\right)=\frac{1}{K}\left|\sum_{k=1}^{K}e^{j\cdot \Delta \phi_{k}\left(f_{m},f_{n},t\right)}\right|.$$

The *m* : *n* PLV can be used to determine the cross-frequency functional connectivity of brain oscillations from distinct frequency bands between two EEG dipoles. The two time courses of a component pair were first bandpass-filtered with a narrow-band convolution filter centered at a specified frequency with a bandwidth of 2 Hz. The PLVs were calculated in every frequency bin from 2 to 50 Hz and at each time point. The PLVs from each pair of component dipoles selected from the frontal, temporal, and parietal regions were then averaged over every 300 ms during memory retrieval or under the condition of distractor trials.

To verify the significance of phase-locking between a pair of components, the signal of components were randomly permuted and surrogate PLVs were generated. The frequency ranges (delta, theta, alpha, and gamma) of the paired components were selected by comparing the observed PLVs with ten surrogate data in the initial stage. After the frequency ranges with pronounced PLVs were decided, the surrogate distribution was constructed using 500 random PLVs. The significant *p*-value associated with an observed PLV was computed by comparing the value with the surrogate distribution.

### Statistical Analysis

Statistical analyses were performed using MATLAB R2013b. The parametric paired *t*-test was conducted to examine the difference of ERSPs between the two experimental conditions. For the analysis of ERPAC and phase-locking, planned comparisons were performed using the surrogate data procedure described in Sections “Event-Related Phase-Amplitude Coupling” and “Phase-Locking Value” to assess physiological measurements under different experimental conditions. According to previous studies (Stam et al., 2007; Axmacher et al., 2010; Foster and Parvizi, 2012; Sun et al., 2012), the significance of phase-locking and phase-amplitude coupling are usually performed using the surrogate test. Group analyses on ERPAC values and PLVs were therefore performed by comparing the data with random-permuted surrogate distributions.

## Results

### Subjective Rating of Music Familiarity and Behavioral Performance in the Musical Delayed Match-to-Sample Task

As mentioned before, of all thirty-six participants recruited for the study, six were rejected due to low task accuracy (i.e., <70%), and another 10 were excluded from further analysis due to their loose dipole fitting. For the remaining 20 participants, their mean reaction time to recognize the main theme in the retrieval phase was 3,865 ms (*SD* = 138 ms), with a mean accuracy of 91% (*SD* = 9.9%). None of the participants were music experts, but most of them had learned at least one instrument systematically for no more than 5 years. In addition, most of the participants reported being in the habit of listening to music for at least 1 h per week.

### Independent Component Clusters

**FIGURE 2:**
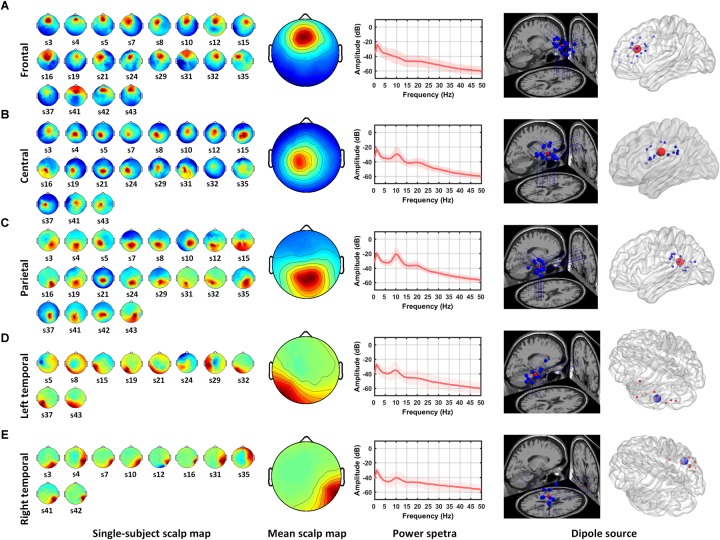
Mean scalp maps and dipole locations of five independent component clusters. The left panel shows the scalp maps found by ICA from individual subjects. The middle panel shows the average scalp map and power spectra from the IC cluster. The right two panels shows the projections of dipole locations on the brain template including five regions: **(A)** frontal, **(B)** central, **(C)** parietal, **(D)** left temporal, and **(E)** right temporal regions.

Five IC clusters were observed from the 20 participants. Individual scalp maps of the frontal, central, parietal, and bilateral temporal regions are illustrated in the left panel of Figure 2A–E. Since participants without frontal or parietal ICs were excluded, the 20 ICs were contributed by all of the participants in their frontal and parietal regions. Only nineteen ICs were contributed by participants in the central region. For the left and right temporal regions, at least one IC from one of the two regions was required from each participant; thus, ten ICs were contributed by each region, respectively. The mean scalp maps, power spectra (middle panel), and dipole locations (the right two panels) from the five IC clusters are also shown in Figure 2. The information about independent components clustered into five brain regions are listed in Table 1, including the Talairach coordinates of the central dipoles, mean residual variance, and number of contributing participants. The average residual variance of all 78 components from the five regions was 6.64%. According to the Talairach coordinates, the five regions were source localized around the anterior cingulate cortex (ACC, Brodmann area 32) in the frontal region, the cingulate gyrus in the central region, the left and right inferior temporal gyrus (ITG, Brodmann area 20 and 35), and the posterior cingulate cortex (PCC, Brodmann area 25) in the parietal region. Similar or close dipole locations have also been reported by previous studies employing auditory tasks (Nolden et al., 2013; Lin et al., 2014; MacLean and Ward, 2014).

**Table 1:** Independent component clusters and the centroid of their source distributions.

Brain region	# of components	Mean residual variance (%)	Talairach coordinates		
			*x*	*y*	*z*
Frontal	20	6.25	−6	27	23
Central	19	5.98	−17	−13	26
Left temporal	9	11.5	−45	−41	−1
Right temporal	10	10.5	52	−53	−9
Parietal	20	3.55	0	−48	21

### Event-Related Spectral Perturbations

**FIGURE 3:**
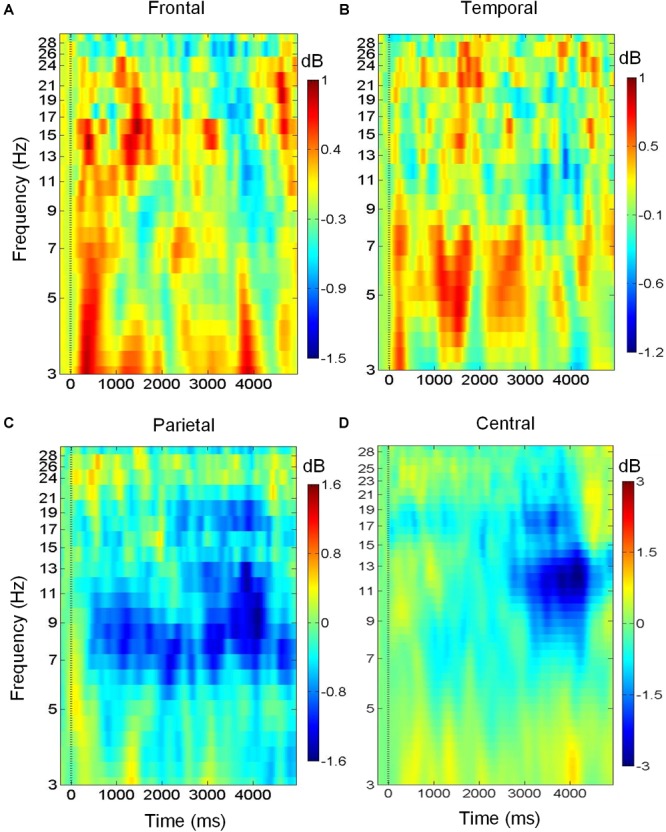
Brain oscillations during musical memory retrieval. **(A)** In the frontal region, there is delta and theta power synchronization during auditory memory retrieval. **(B)** In the temporal region, theta synchronization is observed. **(C)** In the parietal region, alpha desynchronization is found during memory retrieval. **(D)** Alpha and beta desynchronization is observed during the reaction in central region.

Figure 3A–D shows the variation of brain oscillations in the frontal, bilateral temporal, central, and parietal regions during the retrieval phase of musical DMST. Increases and decreases in the power of different frequency bands relative to the pre-stimulus baseline are represented by red and blue colors, respectively. The ERSPs during the retrieval of the targets in the four brain regions are shown in Figure 3. As illustrated in Figure 3A, there were low frequency oscillations in the frontal region during the retrieval of melodies stored in working memory. Delta (1–4 Hz) and theta (4–7 Hz) event-related synchronization (ERS) appeared in the first 1,000-ms window after the stimulus onset, and beta (12–30 Hz) ERS was observed in the first 2,000-ms window. These findings were consistent with previous studies of auditory working memory (Knyazev, 2007; Düzel et al., 2010; Waldhauser et al., 2012; Berger et al., 2014; Chen and Caplan, 2017). Pronounced theta synchronization was also observed in bilateral temporal regions during auditory memory retrieval (Figure 3B). Theta ERS to the target was found in the time interval of 1,000–2000 ms after the stimulus onset, which might indicate the timing of successful identification of the memory items. In contrast, alpha (8–12 Hz) desynchronization was observed in the parietal region during memory retrieval with beta desynchronization found later during motor execution of successful recognition (Figure 3C). In the central region, desynchronization with a spectral peak near 10 and 22 Hz indicates the mu rhythm after movement when the subject identified the main theme during the reaction of memory retrieval (Babiloni et al., 1999). As shown in Figure 3D, ERSPs are time-warped to the average reaction time among all of the participants. The desynchronization observed in the mu frequency band (8–12 Hz) is typically considered evidence of motor preparation (Derambure et al., 1999; Pineda, 2005; Kumar et al., 2013).

### Event-Related Phase-Amplitude Coupling

**FIGURE 4:**
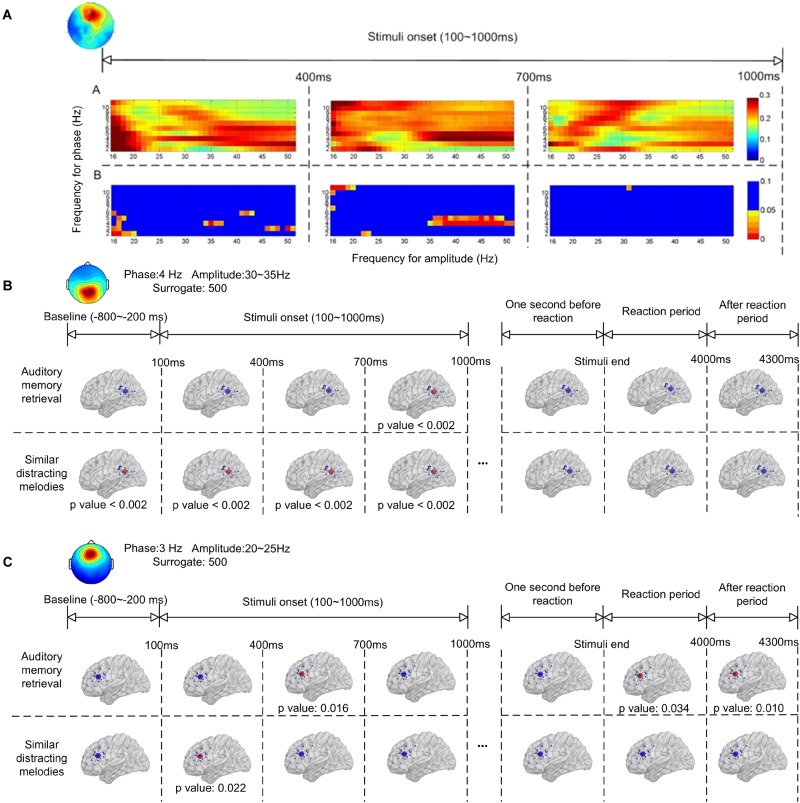
Event-related phase-amplitude coupling (ERPAC) of an independent component located in the frontal region during auditory memory retrieval **(A)**. The upper trace shows the raw ERPAC level, and the lower trace shows the significance using random surrogating. There is significance in theta band between 400–700 ms after the stimulus onset. Group results of ERPAC are shown in the frontal **(B)** and parietal **(C)** regions during memory retrieval and other time periods. There is significance between the delta/gamma coupling in the frontal region and the theta/gamma coupling in the parietal region during memory retrieval after random surrogating of the signals.

Figure 4 shows the ERPAC within the frontal and parietal regions. As shown in Figure 2, the centroids of the source distributions in the frontal and parietal regions were located around the ACC and PCC, respectively. Figure 4A illustrates an example of the cross-frequency ERPAC within the ACC from one of the participants, in which a high level of ERPAC between the signal frequency for phase of 3–5 Hz and the frequency for amplitude of 30–50 Hz was observed in the 400- to 700-ms window after the stimulus onset. The upper trace of Figure 4A shows the raw ERPAC values, and the lower trace shows the significance after surrogation. The higher level of phase-amplitude coupling was mainly observed in theta and low gamma (30–50 Hz) ranges compared with other frequency bands during musical memory retrieval.

The group significance of the ERPAC within the parietal and frontal regions is illustrated in Figure 4B,C, respectively, after applying the *t*-test between group ERPACs and random surrogation 500 times. The largest values of the group-averaged ERPACs were clustered with signals filtered and surrogated again. As shown in Figure 4B, the pronounced ERPACs observed in the parietal region were between the frequency for phase of 4-Hz EEG signals and the frequency for amplitude of 30–35 Hz, illustrating the theta–gamma ERPAC within the parietal region close to the PCC. Compared with the baseline (800–200 ms before the onset of stimuli) which showed no significance, the upper trace of Figure 4B shows the significance of theta–gamma ERPAC between 700 and 1000 ms (*p* < 0.002) during memory retrieval. In contrast, pronounced theta–gamma ERPAC was found in the distractor trials with similar melodies (lower trace of Figure 4B) in the first 1,000 ms after the onset of stimuli (*p* < 0.002), whereas the significance was also observed before the stimulus onset. The ERPAC results also suggested that there was increased delta and high beta (20–25 Hz) cross-frequency ERPAC in the frontal region close to the ACC during memory retrieval (Figure 4C). Pronounced ERPACs were found during memory retrieval at between 400 and 700 ms (*p* = 0.016) and also, during distractor trials, in the first 400 ms (*p* = 0.022) after the stimulus onset.

### Functional Connectivity

**FIGURE 5:**
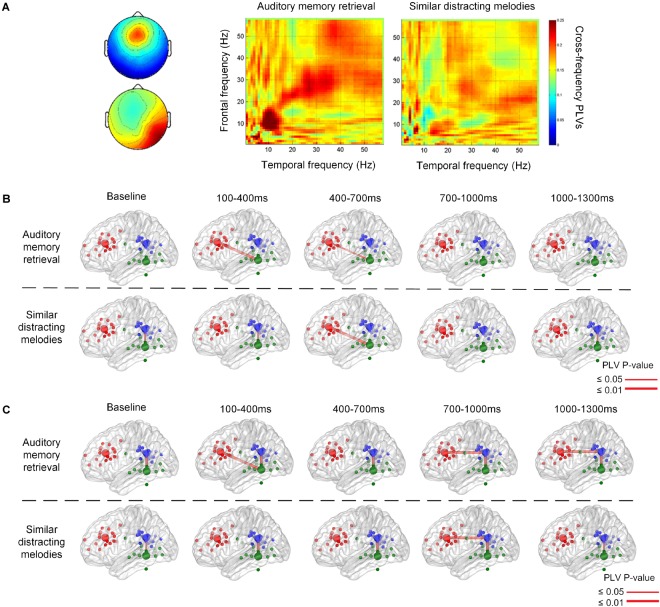
An example of cross frequency phase lock value between 100 and 400 ms after the stimulus onset **(A)**. The phase synchronization between the frontal and temporal regions during memory retrieval and similar distracting conditions. Group results of phase-locking values are illustrated between the frontal, parietal, and temporal regions in theta **(B)** and alpha **(C)** frequency bands. There is theta connection between the frontal and temporal regions during auditory memory retrieval, and there is also alpha correlation between the frontal and parietal regions during the memory retrieval of targets and distractors.

Figure 5 illustrates the phase synchronization among the frontal, temporal, and parietal regions. An example of the cross-frequency PLVs between the frontal IC close to ACC and right temporal IC close to ITG of a single subject (Figure 5A) shows that higher level PLVs were observed during the retention of auditory memory, compared with the distractor trials with similar melodies. The higher level of phase synchronization was mainly observed in theta and alpha during natural auditory memory retrieval.

The group significance of phase synchronization among the frontal, parietal, and left or right temporal regions is illustrated in Figure 5B,C after the *t*-test between group PLVs and random surrogation 500 times. Theta phase synchronization between the frontal and posterior regions was observed during the first 100–1,000 ms during auditory memory retention, as shown in the upper row of Figure 5B. Although the main theme lasted for 3,896 ms, phase synchronization between the frontal and temporal regions was found in the very beginning during the first 700 ms (*p* = 0.010 between 100 and 400 ms, and *p* = 0.038 between 400 and 700 ms). In contract, distractor trials showed more pronounced fronto-temporal phase synchronization (*p* = 0.010) in the theta frequency band 400 ms after the stimulus onset (the lower trace in Figure 5B), and the high level of PLVs lasted for at least 900 ms. In addition, the phase synchronization between fronto-temporal and fronto-parietal regions were not found in the baseline, which was 800–200 ms before the onset of each stimulus. The phase synchronization between the frontal and parietal regions was observed in the alpha frequency band after 700 ms (*p* < 0.002), as denoted in Figure 5C. The synchronization lasted for a longer time period (600 ms) during auditory memory retrieval. Consistent temporal-parietal phase-locking was observed in the alpha band (*p* < 0.002) during auditory memory retrieval of the two conditions, as well as during the baseline periods. In summary, increased phase synchronization in theta frequency was observed between the frontal and temporal regions, and there was also alpha-band correlation between the fronto-parietal during the auditory memory retrieval and disturbance trials of similar melodies. The results also indicated that the temporal dynamics of phase synchronization that increased fronto-temporal PLVs (theta band, 100–700 ms) were observed earlier than fronto-parietal PLVs (alpha band, after 700 ms).

## Discussion

The aim of this study was to explore the neural dynamics contributing to musical working memory with our newly established natural musical DMST. Consistent with previous EEG and MEG findings in auditory working memory (Nolden et al., 2013; Lin et al., 2014; MacLean and Ward, 2014), our ICA and dipole fitting results showed elevated activation in corresponding regions, such as the left and right inferior temporal gyrus, anterior cingulate cortex, and posterior cingulate cortex, which have been reported to be correlated with the retention of auditory short-term memory, the perception of retention, and memory load, respectively (Nolden et al., 2013). We also demonstrated the following findings. First, low-frequency oscillations in the frontal and temporal regions were found to be increased during the retrieval phase of the musical DMST. Second, the PAC between the low-frequency phase and amplitudes of the higher frequency band was observed in the frontal and parietal regions in the first 1,000 ms interval of memory retrieval. This outcome suggests that the PAC in these regions might serve as a neural mechanism contributing to the retrieval and identification processes of musical working memory. Third, the results of phase synchronization or phase-locking revealed that there was pronounced fronto-temporal communication in the theta band and fronto-parietal communication in the alpha band with the presentation of both the target and distracting melodies, and their onset was earlier with the target than with the distractors. Detailed findings and their connections with previous studies are discussed in the following subsections, and we look forward to drawing a precise picture of the neural dynamics underlying musical working memory.

### Low-Frequency Power Synchronization in Fronto-Temporal Regions and Alpha Desynchronization in Parietal Region

Our ERSP results showed that there was pronounced delta and theta power synchronization between the frontal ACC and temporal ITC regions in the first 1,000-ms interval of stimulus presentation during the retrieval phase. Theta synchronization has long been reported to be an indicator of visual working memory, especially the retrieval process (Klimesch, 1999; Knyazev, 2007; Düzel et al., 2010). Previous studies using episodic memory tasks (Waldhauser et al., 2012) and recognition memory tasks (Chen and Caplan, 2017) have found that frontal theta power is increased with the successful retrieval of working memory. In contrast, the successful suppression of target visual memories is reflected by a decrease in theta oscillations (Waldhauser et al., 2014). Importantly, in the auditory modality, studies have paid attention to change detection, and corresponding neural networks have identified increased theta synchronization in fronto-temporal regions (Hsiao et al., 2009; MacLean and Ward, 2014). Furthermore, Berger et al. (2014) adopted a verbal delayed DMST and found a linear increase in frontal-midline theta relative to task load. Hence, consistent with previous findings, the frontal and temporal synchronization observed in the present study reflected the retrieval processes of musical working memory.

In addition, we also obtained significant alpha power desynchronization of the presentation of target melodies. In previous studies, alpha desynchronizations in thalamo-cortical feedback loops (Klimesch, 1999) and posterior brain areas (Klimesch et al., 1996; Klimesch and Schack, 2003) were reported to be correlated with searching and retrieval performance in semantic long-term memory tasks. In contrast, alpha activity increases if the task condition does not require access to long-term memory (Klimesch et al., 2007; Berger et al., 2014). Additionally, studies focusing on the relationship between alpha oscillations and expectations about the temporal occurrence of stimuli in the central and parietal regions (Wilsch et al., 2014, 2015; Wöstmann et al., 2015) have reported that alpha activity increases with the cognitive load in memory retention tasks (Leiberg et al., 2006; Wilsch and Obleser, 2016), and it decreases when stimuli are temporally expected. Thus, we suggest that alpha desynchronization to the target melodies in our study indexed a matching and identification process.

### Event-Related Phase-Amplitude Coupling in Frontal and Parietal Brain Regions

Following the findings of low-frequency power synchronization in the frontal, temporal, and parietal regions (Figure 3), we further identified the increase in PAC, which has been considered an indicator of the engagement of several cognitive functions (Canolty et al., 2006; Lisman and Buzsáki, 2008; Tort et al., 2009; Axmacher et al., 2010; Friese et al., 2013; Lega et al., 2014; Roux and Uhlhaas, 2014; Voytek et al., 2015; Park et al., 2016; Rajji et al., 2016). Our results of ERPAC showed that there was increased theta phase and gamma amplitude ERPAC in the parietal region during the retrieval phase of musical DMST, as well as delta phase and beta amplitude ERPAC in the frontal region. Coupling between the phase and amplitude of different frequency bands has been observed in experiments, such as visual short-term memory (Axmacher et al., 2010; Friese et al., 2013; Lega et al., 2014; Roux and Uhlhaas, 2014; Voytek et al., 2015; Park et al., 2016; Rajji et al., 2016), associative learning (Lisman and Buzsáki, 2008; Tort et al., 2009, 2010), and other memory-related tasks (Canolty et al., 2006; Cohen et al., 2009; Handel and Haarmeier, 2009; Voytek et al., 2010; Arnal et al., 2014). For theta–gamma PAC, it was first reported in animal experiments of spatial learning and memory (Lisman and Jensen, 2013). Consistent findings of theta–gamma couplings were later verified in the human and animal hippocampus during a variety of cognitive tasks (Lisman and Buzsáki, 2008; Tort et al., 2009; Axmacher et al., 2010; Tort et al., 2010; Voytek et al., 2010). These high-frequency gamma waves serve as clocks or temporal references for the theta phase code during encoding or retrieving of discrete items, especially in the place cells of the hippocampus (Lisman and Jensen, 2013). Recent studies have further claimed that this phenomenon can also be observed using EEG and MEG, supporting the engagement of the neocortex (Cohen et al., 2009; Handel and Haarmeier, 2009; Axmacher et al., 2010; Friese et al., 2013; Arnal et al., 2014; Lega et al., 2014; Roux and Uhlhaas, 2014; Park et al., 2016; Rajji et al., 2016). However, when the PAC is manifested in the neocortex, the coupling frequency bands might not be restricted to theta–gamma pairs. According to the results listed in Table 2, which summarizes the modalities, involved brain regions, and frequency bands of PAC, it could be observed that all of the studies recording local field potential (LPF) from the hippocampus reported precise coupling between the theta phase and gamma amplitude. In contrast, many of the EEG and MEG studies have reported coupling from different frequency bands. Our EEG results also indicated that there was PAC in both the frontal and parietal regions during musical DMST, and coupling was observed between theta/gamma and delta/beta frequency bands. In addition, a large between-subject variation in the coupling frequency bands was observed, although significance was achieved. In agreement with the phenomenon reported by Lisman and Jensen (2013), we suggest that the precise PAC observed in the hippocampus with a modulating phase in the theta band could be redefined as launched low-frequency oscillations after propagating to the neocortex, possibly engaging delta, theta, and alpha waves. Simultaneous recording of fMRI BOLD responses and EEG reactions also found that theta and alpha power correlates with the hippocampal connection to the prefrontal cortex and striatum in a recognition memory task, suggesting that the theta oscillation directs information flows in hippocampal networks by coupling with higher frequencies such as alpha activities in different stages of memory formation (Herweg et al., 2016).

**Table 2:** Previous findings of PACs in distinct imaging modalities, cognitive tasks, and the involved brain regions.

Study	Subject	Task	Modality	Brain regions	Coupling frequency band
Canolty et al., 2006	Human	Cognitive tasks	ECoG	Frontal Temporal	Theta/gamma
Lisman and Buzsáki, 2008	Rat	Learning	LFP	Hippocampus	Theta/gamma
Cohen, 2008; Cohen et al., 2009	Human	Reward	iEEG	Frontal	Alpha/gamma
Tort et al., 2009	Rat	Learning	LFP	Hippocampus	Theta/gamma
Handel and Haarmeier, 2009	Human	Visual sensory	MEG	Frontal Parietal	Delta/gamma
Voytek et al., 2010	Rat	Visual	ECoG	Hippocampus	Theta/gamma
Axmacher et al., 2010	Human	Working memory	iEEG	Hippocampus	Theta/gamma
Tort et al., 2010	Rat	Learning	LFP	Hippocampus	Theta/gamma
Friese et al., 2013	Human	Memory encoding	EEG	Frontal Posterior	Theta/gamma
Roux and Uhlhaas, 2014	Human	Working memory	EEG	Frontal Parietal	Theta/alpha Alpha/gamma
Arnal et al., 2014	Human	Temporal prediction	MEG	Frontal Parietal	Delta/beta
Lega et al., 2014	Human	Memory formation	ECoG	Frontal Temporal	Theta/gamma Alpha/gamma
Voytek et al., 2015	Human	Information encoding	ECoG	Prefrontal	Theta/gamma
Rajji et al., 2016	Human	Working memory	EEG	Frontal	Theta/gamma
Park et al., 2016	Human	Visual memory	MEG	Visual cortex	Alpha/gamma
Our results	Human	Auditory memory	EEG	Frontal Parietal	Delta/beta Theta/gamma

*LFP, local field potential; ECoG, electrocorticography; iEEG, intracranial EEG.*

Another interesting finding was that the PAC was also observed during the distracting melodies (Figure 4). Additionally, we evaluated the temporal dynamics of PAC during memory retrieval using ERPAC (Voytek et al., 2013), and found that it was also pronounced at the very beginning of the presentation of the distractors (Figure 4C), when the melodies were identical to the targets and could not yet be distinguished. However, the results of theta PAC showed that the increased PAC could also be observed in the baseline periods (Figure 4B), which made it difficult to discriminate between memory retrieval and musical perception. Since the effect of distraction or competing stimuli on working memory has yet to be correlated with PAC, further investigation is still demanded. In spite of this, some recent studies have reported that the PAC is positively correlated with working memory load and with the allocation of cognitive resources (Axmacher et al., 2010; Rajji et al., 2016). As for our results, recognizing and distinguishing similar distracting melodies from the target would result in a greater processing load and would require more resources, leading to an increase in PAC.

### Fronto-Temporal and Fronto-Parietal Phase-Locking in Theta and Alpha Frequency Bands

In contrast to the PAC, which is usually applied to assess intra-regional activities, phase-locking has often been reported for evaluating inter-regional communications. Brain connectivity is another important approach for accessing the functional pathways of memory processes. There have been many studies discussing functional connectivity of visual working memory (Von Stein and Sarnthein, 2000; Sauseng et al., 2004, 2005; Kawasaki et al., 2014). In comparison, the pathway of auditory working memory has only recently begun to receive some attention during this decade. Previous studies conducting visual working memory tasks often examined the involvement of networks among the frontal, parietal, and temporal regions, especially coherence in theta and alpha bands (Sauseng et al., 2004, 2010). Sauseng et al. (2004) reported the theta coupling between the frontal and temporal regions during memory encoding, whereas frontal and bilateral temporal-parietal couplings have been observed in retrieval. In addition, the fronto-parietal EEG coherence in theta and alpha was claimed to reflect central executive functions of working memory (Sauseng et al., 2005). Later, Fell et al. (2008) and Fell and Axmacher (2011) utilized phase-locking within the human medial temporal lobe on intracranial EEG to predict memory formation. Furthermore, recent studies have reported that not only in memory encoding but also the maintenance of memory (Toth et al., 2012) and working memory capacity (Gulbinaite et al., 2014) are reflected the increases in delta and theta coupling between the frontal and parietal regions. The role of phase-locking in memory processes might result from neural communication, plasticity, or coincidence detection (Fell and Axmacher, 2011).

Unlike visual memory, the map of the relationship between auditory memory processes and brain connections is still under construction. Our phase-locking results have suggested that there is an increased fronto-parietal and parietal-temporal connection in the alpha band during musical DMST, which is in agreement with previous studies of visual memory, shown in Table 3 (Von Stein and Sarnthein, 2000; Sauseng et al., 2005; Toth et al., 2012; Gulbinaite et al., 2014; Kawasaki et al., 2014). Another finding of this study was the phase-locking of the theta band between the frontal and temporal regions at the beginning of memory retrieval and with distracting melodies. In auditory research, fronto-temporal phase synchronization has consistently been found in auditory oddball tasks. Lin et al. (2007) reported enhanced phase synchronization between the frontal and temporal regions in auditory oddball MEG signals in the time frame of 150–250 ms after the stimulus onset. Later, Hsiao et al. (2010, 2014) reported the neuronal network between frontal and temporal regions in theta and alpha bands during auditory oddball tasks. Temporo-frontal, temporo-parietal, and within-temporal neuronal networks are related to detection of auditory changes (Hsiao et al., 2010). Consistent results have also been reported by Choi et al. (2013), and also MacLean and Ward (2014), supporting the hypothesis that frontal and temporal interactions are important during auditory deviant processing. Among all of the previous studies, only two of them illustrated auditory memory (Von Stein and Sarnthein, 2000; Kawasaki et al., 2014), as shown in Table 3. In this study, we found consistent results of temporo-parietal alpha phase synchronization in both musical delayed DMST and basic auditory perception during naturalistic music listening. The phase-locking of fronto-temporal in the theta band and fronto-parietal in the alpha band were found in the musical delayed DMST during memory retrieval. Although the memory item in this task, which was a 3,896-ms main theme, was designed to be longer than those items in previous studies, the pronounced phase synchronization was still within the first 1,000 ms of memory retrieval. Furthermore, the connection was observed under task conditions with both target and distracting melodies.

**Table 3:** Previous findings of PLVs in distinct imaging modalities, cognitive tasks, and the involved brain regions.

Study	Task	Modality	Brain connectivity	Frequency band
Von Stein and Sarnthein, 2000	Visual and auditory working memory	EEG	Parietal-Temporal Frontal-Parietal	Beta, theta, alpha
Sauseng et al., 2004	Visual working memory	EEG	Frontal-Temporal	Theta (0–500 ms)
Sauseng et al., 2005	Visual working memory	EEG	Frontal-Parietal	Theta, alpha
Hsiao et al., 2010	Auditory MMN	MEG	Frontal-Temporal Parietal-Temporal	Theta, alpha (150–300 ms)
Toth et al., 2012	Visual memory maintenance	EEG	Frontal-Temporal Frontal-Parietal	Delta, theta
Choi et al., 2013	Auditory MMN	MEG	Frontal-Temporal	Theta (100–300 ms)
Kawasaki et al., 2014	Visual and auditory-verbal working memory	EEG	Frontal-Temporal Frontal-Parietal	Theta
Gulbinaite et al., 2014	Visual working memory capacity	EEG	Frontal-Parietal	Delta, theta
MacLean and Ward, 2014	Auditory MMN	EEG	Frontal-Temporal	Theta, gamma (120–250 ms)
Albouy et al., 2017	Auditory working memory	MEG	Frontal-Parietal Bilateral Parietal	Theta
Our results	Musical delayed match-to-sample	EEG	Frontal-Temporal Frontal-Parietal Parietal-Temporal	Theta (100–700 ms) Alpha (700–1,300 ms)

*MMN, mismatch negativity.*

Despite the limitation of a small sample size, this study included a cohort of participants for the experiment and used a high-resolution EEG system to record the data. By carefully adjusting EEG artifacts, the preprocessed data were analyzed with different methods. The results from behavioral data, ERSPs, ERPACs, and phase synchronization reached convergent findings to study the pathways of auditory working memory. Furthermore, the design of unpredictable and naturalistic musical DMST could minimize the slow brain rhythms possibly generated by a predictable paradigm with a regular rhythm of the occurrence of stimuli, thus preventing the enhancement of low-frequency signals and biased results in memory-related cognitive studies (Schroeder and Lakatos, 2009). The naturalistic paradigm reported in this study might improve the reliability of observed brain responses, especially regarding the analysis of ERSPs, ERPACs, and PLVs focusing on brain oscillations between frequency bands. These convergent findings, without a large between-subject difference, render the utilization of complex and naturalistic stimuli more convincing for application in real-life settings (Hasson et al., 2010).

## Conclusion

We designed an experimental paradigm of memory retrieval with natural auditory stimulation. The timing, intra- and inter-regional correlations of the reported musical DMST were discussed among the frontal, temporal and parietal brain regions. The results provided a more precise timeline to evaluate the activation, phase-amplitude coupling, and functional connectivity, and suggested that brain oscillations and couplings occur at the beginning of musical DMST. Furthermore, the activation and coupling were also pronounced during the distracting melodies, which could indicate that memory resources are also involved in the rejection of similar distractions. This study demonstrated the possibility of using natural auditory stimuli as an experimental paradigm, which is closer to real life, and thus increases the possible applications of the EEG paradigm for evaluating the effectiveness of treatments, a training paradigm, or music therapy for participants with mental disorders.

## Ethics Statement

This study was carried out in accordance with the recommendations of the human subject research ethics committee/Institutional Review Board (IRB) at the Academia Sinica, Taiwan, with written informed consent from all subjects.

## Author Contributions

Y-LT, H-HL, and ML conceived analytical hypothesis, performed data analysis and interpretation, and drafted and revised the work. AT, VC, S-TS, and Z-SY performed data analysis and interpretation. All authors approved the work for publication.

## Conflict of Interest Statement

The authors declare that the research was conducted in the absence of any commercial or financial relationships that could be construed as a potential conflict of interest.
